# Case Report: Mucolipidosis II and III Alpha/Beta Caused by Pathogenic Variants in the *GNPTAB* Gene (Mucolipidosis)

**DOI:** 10.3389/fped.2022.852701

**Published:** 2022-04-08

**Authors:** Shao-Jia Mao, Yu-Mei Zu, Yang-Li Dai, Chao-Chun Zou

**Affiliations:** Department of Endocrinology, The Children's Hospital of Zhejiang University School of Medicine, Hangzhou, China

**Keywords:** *GNPTAB*, *N*-acetylglucosamine-1-phosphotransferase, mucolipidosis, genetic testing, variant site

## Abstract

**Objective:**

This study aimed to improve the cognition of mucolipidosis (ML) II and III alpha/beta by analyzing the clinical manifestations of two patients.

**Methods:**

The clinical, biochemical, and molecular data of two clinical cases associated with ML II and III alpha/beta were analyzed and compared with other case reports of ML II and III alpha/beta.

**Results:**

The first patient was a 14-month-old girl who was hospitalized because of abnormal postnatal coarse facial features. The child had no abnormal birth history, but developed multiple abnormalities such as psychomotor retardation, abnormal facial features, bilateral limb muscle hypotonia, and genital abnormalities. The X-ray of the spine revealed multiple bone malformations. Brain magnetic resonance imaging (MRI) showed delayed myelination. Genetic testing showed the presence of two compound heterozygous pathogenic variants (c.1364C>T and c.1284+1G>T) in the *GNPTAB* gene. The second patient was an 18-month-old boy who was hospitalized for recurrent respiratory tract infections. The patient was a high-risk preterm infant with postnatal psychomotor retardation, language development retardation, intellectual disability, and coarse facial features. X-ray showed multiple bone malformations. Craniocerebral ultrasound showed bilateral ventricle widening. Genetic testing showed the presence of two compound heterozygous pathogenic variants (c.1284+1G>T and c.483delT) in the same gene.

**Conclusions:**

ML II and III alpha/beta are rare autosomal-recessive lysosomal storage diseases that are attributed to *GNPTAB* variants that cause *N*-acetylglucosamine-1-phosphotransferase deficiency, finally leading to multiple clinical signs and symptoms. A proper ML II and/or III alpha/beta diagnosis requires a combined analysis of a patient's clinical manifestations, imaging examination, enzymatic analysis, and genetic testing results. Ultimately, genetic counseling is essential for this disease.

## Introduction

Mucolipidosis (ML) II (OMIM 252500) and ML III (OMIM 252600) alpha/beta are lysosomal storage diseases (LSDs) caused by variants in the *GNPTAB* gene located on chromosome 12q23.2. The occurrence of these two diseases is a consequence of autosomal-recessive inheritance. The *GNPTAB* gene encodes the alpha and beta subunits of *N*-acetylglucosamine-1 (GlcNAc)-phosphotransferase (EC 2.7.8.17), which is the key enzyme in the synthesis of mannose 6-phosphate (M6P). M6P deficiency makes lysosomal enzymes unable to reach the lysosomes; therefore, a large number of lysosomal enzymes are secreted outside cells, causing an excessive accumulation of lysosomal substrates *in vivo* and various clinical symptoms in multiple organs (1).

The symptoms of ML II and III alpha/beta, including growth retardation, psychomotor retardation, mild cognitive impairment, dysostosis multiplex, recurrent respiratory tract infections, coarse facial features, visceral swelling, heart disease, and gingival hyperplasia, are similar to those observed in some LSDs, such as mucopolysaccharidosis (MPS) and fucosidosis. The major difference between ML II and ML III alpha/beta is that ML II results from a complete loss of GlcNAc-phosphotransferase activity, while ML III results from a partial deficiency of the same enzyme (2). Therefore, patients with ML II alpha/beta are more severe and have an earlier disease onset than those with ML III alpha/beta. The clinical manifestations of ML II alpha/beta usually present at birth or in early infancy. Patients with this disease often die in childhood and generally survive no more than 7 years old (3). The clinical manifestations of ML III alpha/beta generally appear at about 3 years of age. The disease progression is slow with relatively mild symptoms, and most patients with ML III alpha/beta can survive into adulthood (4).

ML II and III alpha/beta are extremely rare diseases with estimated prevalence rates of 0.16–0.8/100,000 and 0.08–1.89/100,000 live births, respectively (5). Hence, most pediatricians have limited knowledge of these diseases, which leads to delays in diagnosis. We report two cases of ML: one diagnosed with ML II and the other with ML III.

## Case Report

### Case 1

A 14-month-old girl was referred to our hospital because of postnatal abnormal facial features. She was G3P2 (third pregnancy and second delivery) of non-consanguineous healthy parents, full term, and was delivered by cesarean section because of little amniotic fluid. The patient weighed 2.4 kg (SD = −1.94) and had a head circumference of 32 cm (SD = −2.35) at birth. Neonatal screening tests, including thyroid-stimulating hormone (TSH) and 17α-hydroxyprogesterone, were all normal. The child was born with neonatal pneumonia. Prior to visiting our hospital, she showed unstable head erection, was unable to raise her head when lying prone, was unable to support the weight of her lower limbs, showed decreased muscle tension and reflex of the limbs, and could not easily laugh out loud. At 4 months old, low free thyroxine (9.9 pmol/L, normal range = 11.5–22.74 pmol/L) and normal TSH (3.22 nmol/L, normal range = 0.93–3.70 nmol/L) were noted. The patient was suspected with central hypothyroidism, so 12.5 μg levothyroxine per day was administrated. No similar genetic disorders were found in her family. After a period of rehabilitation treatment, the patient had no obvious improvement. Her parents and sister were healthy.

**Figure 1:**
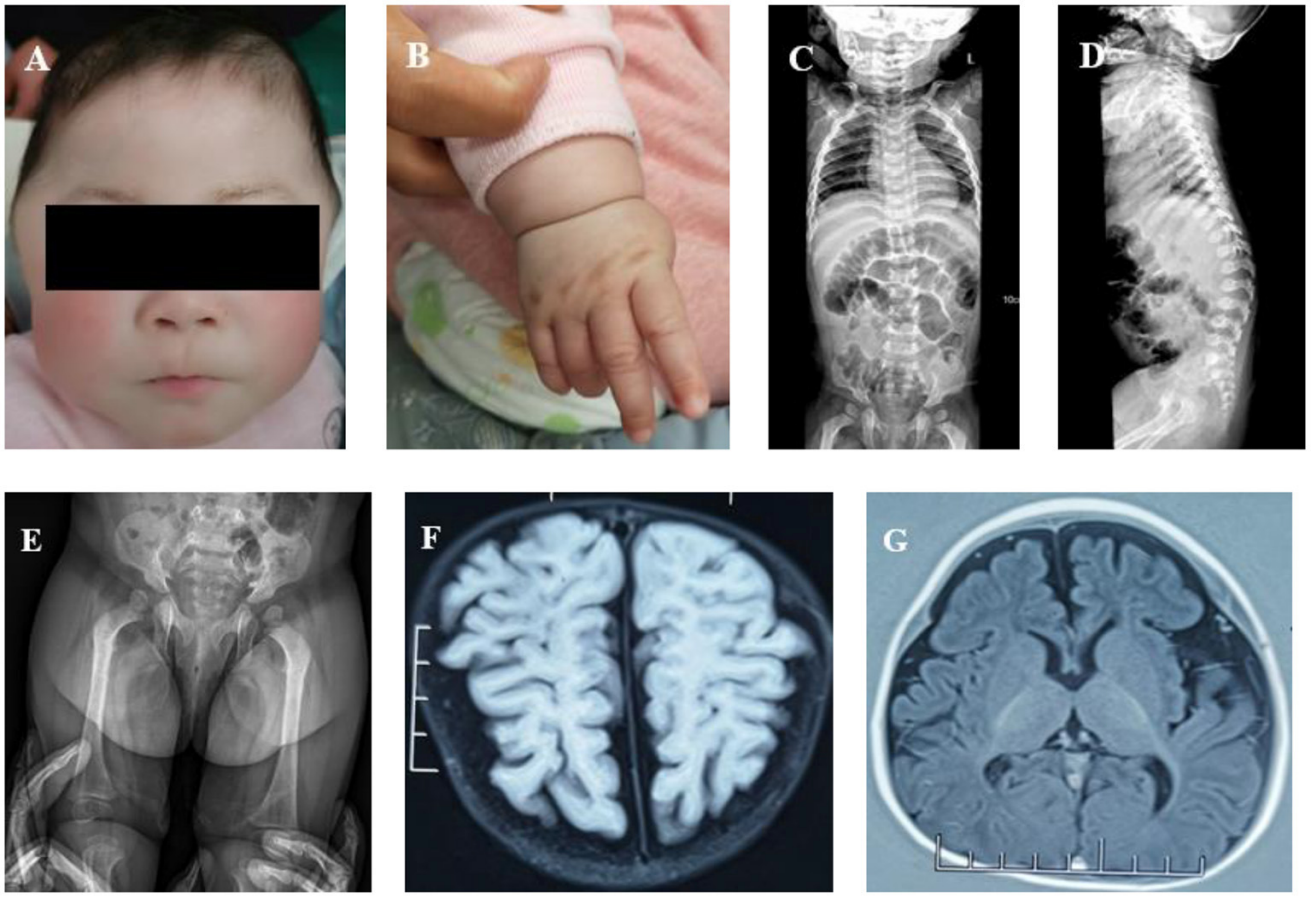
**(A)** Abnormal facial features (a closed anterior fontanelle, prominent temporal parts, long eyelashes, sparse hair, upturned nostrils, a sharp beak-like mouth, low-set ears, a short neck, and white skin). **(B)** Wrist joint seam showing significant stenosis. **(C)** Spine X-ray: slight scoliosis of the spine and slightly narrow vertebral ends of ribs. **(D)** Spine X-ray: the spine showed slight kyphosis at the junction of the thoracolumbar segment, had short anterior and posterior diameters of some thoracolumbar vertebra, and there was anterior breakage of the vertebral body. **(E)** Spine X-ray: irregular acetabulum and lower edge of iliac bone. **(F)** Brain MRI: poor white matter development. **(G)** Brain MRI: smaller frontal lobe and thinner corpus callosum.

Physical examination showed a height of 64 cm (SD = −4.54), a weight of 7.3 kg (SD = −2.31), and a head circumference of 42 cm (SD = −3.93). She had a closed anterior fontanelle, prominent temporal parts, long eyelashes, sparse hair, upturned nostrils, a sharp beak-like mouth, low-set ears, a short neck, and white skin (Figure 1A). The external genitalia was childish, and the wrist joint seam had significant stenosis (Figure 1B). No obvious cleft hand deformity or finger contracture was noted. Her bilateral limb muscle tension was decreased. The patient had harsh breath sounds in both lungs without obvious rales or heart murmur. Hepatosplenomegaly was not examined in this patient. No other obvious abnormality was observed.

The blood cell count, urine routine analysis, liver and kidney functions, and urinary glycosaminoglycan (GAG) screening by dimethylmethylene blue dye binding (DMB) assay were all normal or negative. The X-ray of the spine indicated slight scoliosis (Figure 1C). At the junction of the thoracolumbar segment, the spine showed slight kyphosis. The anterior and posterior diameters of some thoracolumbar vertebrae were smaller than normal, and the anterior edge of the vertebral body presented a beak shape. The shape of the vertebral ends of the ribs was slightly narrow and that of the lateral ends was slightly wide (Figure 1D). The shape of the acetabulum and lower edge of the iliac bone was irregular, and the shape of the femoral neck was thin (Figure 1E). Echocardiography showed a slightly thickened aortic valve with incomplete opening and mild aortic and mitral regurgitation. No hepatosplenomegaly was found by abdominal B-ultrasound. Brain MRI showed less white matter, smaller frontal lobes, and a thinner corpus callosum (Figures 1F,G). The Peabody Developmental Motor Scales indicated that the patient's motor development was obviously retarded. The equivalent age of the patient's reflexes, stationary, locomotion, object manipulation, grasping, and visual–motor integration was 1 month. The total score was under the 5th percentile.

### Case 2

An 18-month-old boy visited our hospital presenting with shortness of breath and cough. He was G1P1 (first pregnancy and first delivery) of non-consanguineous healthy parents and was born at 35 weeks by cesarean section, with a birth weight of 1.8 kg (SD = −3.01) and a head circumference of 30 cm (SD = −4.66). The patient was hospitalized after birth because of neonatal unconjugated hyperbilirubinemia, neonatal hyaline membrane disease, and apnea. At the age of 18 months, he was still unable to raise his head, turn over, sit, crawl, or stand, and his attempted movements were accompanied by a significant increase in muscle tension in both limbs. His language development was slow, and he still could not produce meaningful words. The growth rate was slow. At 18 months, his height was only 65 cm, far shorter than his peers. The patient had recurrent respiratory tract infections and was hospitalized for acute severe pneumonia at the age of 6 and 18 months. He developed bilateral inguinal hernia at 3 months and underwent bilateral high ligation of hernia at 6 months. No similar genetic family history was found.

**Figure 2:**
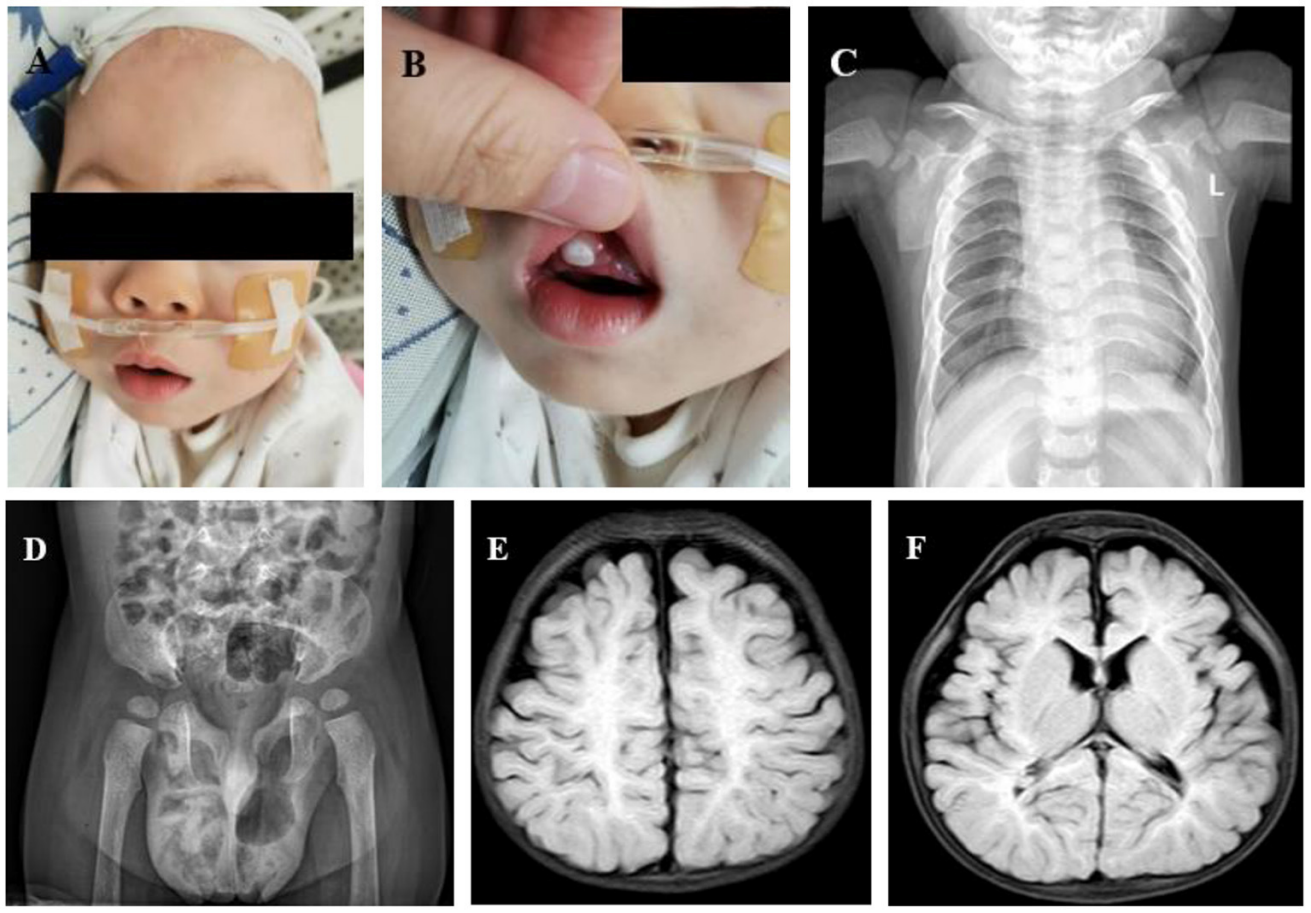
**(A)** Abnormal facial features (thick arched eyebrows, a flat nose, swollen eyelids, exophthalmos, epicanthus, low-set ears, and a short neck). **(B)** Obvious gingival hyperplasia. **(C)** Anteroposterior chest X-ray: small chest shape, short floating ribs, and irregular bilateral proximal humerus. **(D)** Pelvis anteroposterior X-ray: sharp and deep bilateral sciatic incisions; small and shallow bilateral acetabulums. **(E)** Brain MRI: small bilateral frontotemporal lobes, wide extracerebral space, and less white matter. **(F)** Brain MRI: thinner corpus callosum.

**Figure 3:**
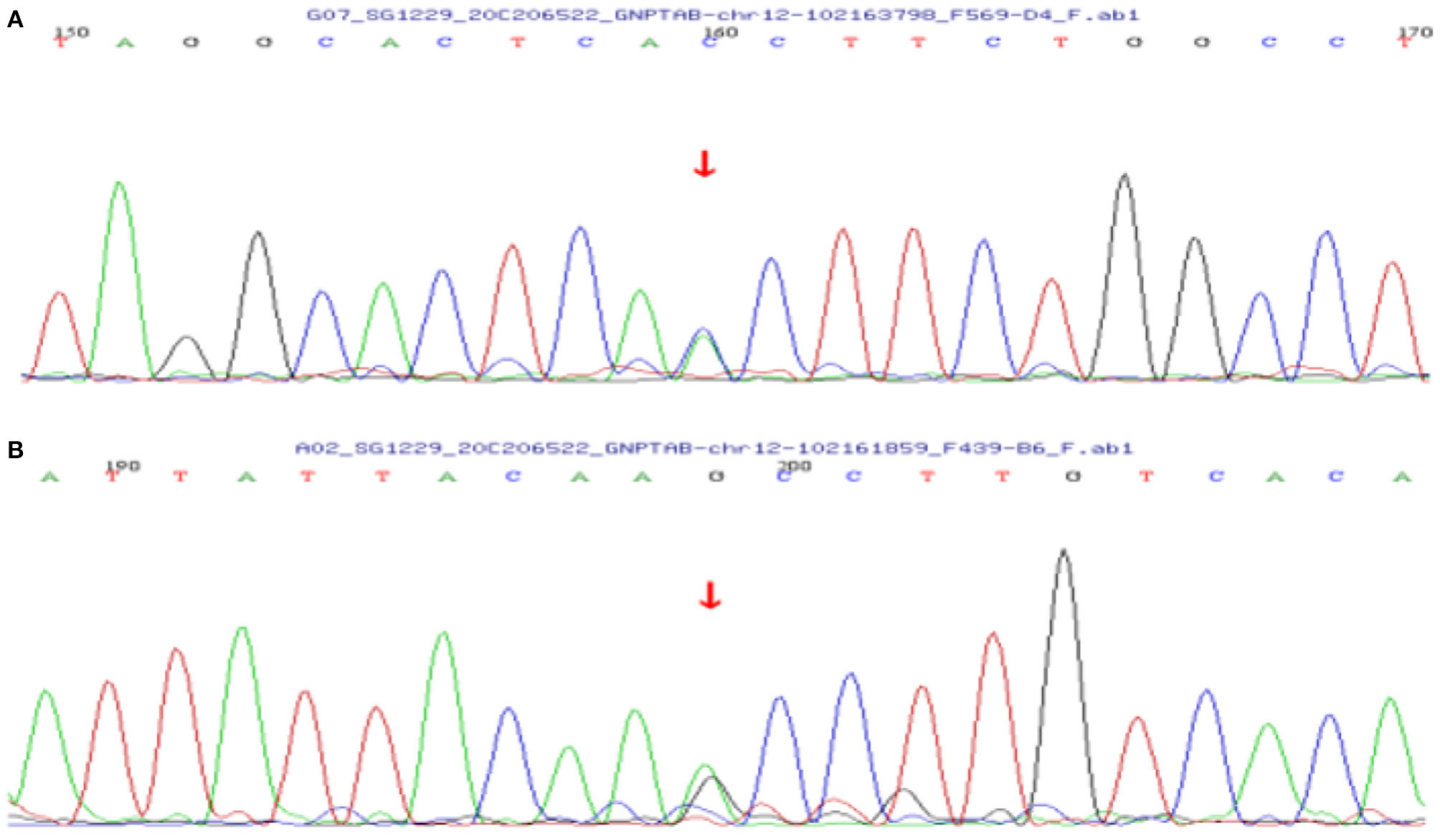
Sanger sequencing chromatograms of the *GNPTAB* gene variants in case 1. **(A)** Heterozygous c.1284+1G>T variant inherited from the mother in the *GNPTAB* gene of the patient. **(B)** Heterozygous c.1364C>T variant inherited from the father in the *GNPTAB* gene of the patient.

**Figure 4:**
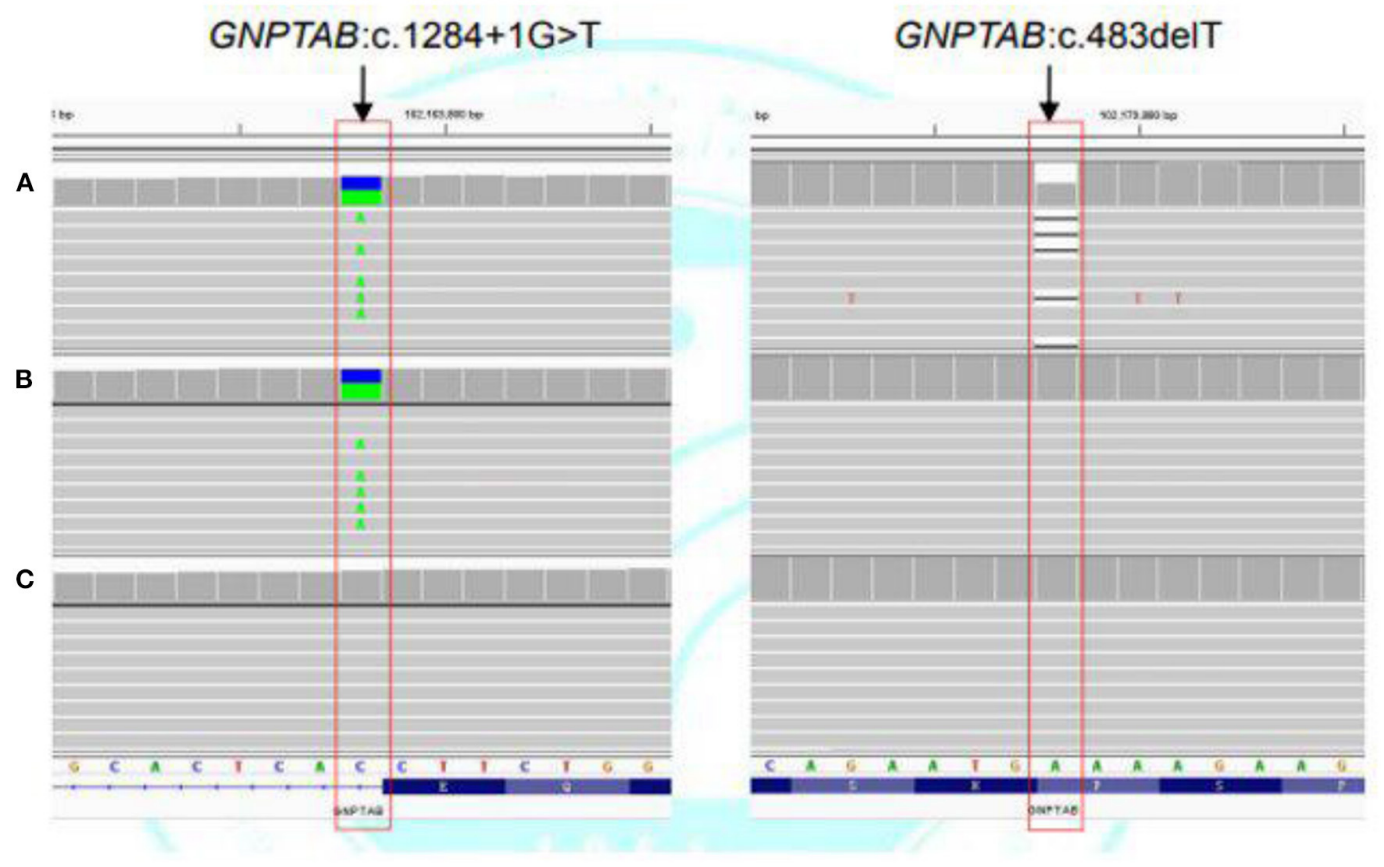
Next-generation sequencing of the *GNPTAB* gene variants in case 2. **(A)** Heterozygous c.1284+1G>T and c.483delT variants in the *GNPTAB* gene of the patient. **(B)** Heterozygous c.1284+1G>T variant in the *GNPTAB* gene of the mother. **(C)** No pathogenic variant was detected in the *GNPTAB* gene of the father.

Physical examination showed a height of 65 cm (SD = −5.74), a weight of 6.5 kg (SD = −4.36), and a head circumference of 41 cm (SD = −6.45) at 19 months. He had thick arched eyebrows, a flat nose, swollen eyelids, exophthalmos, epicanthus, obvious gingival hyperplasia, low-set ears, a short neck, pectus carinatum, short fingers, and stiff joints (Figures 2A–**4B**). The anterior fontanelle was closed early at 9 months. Limb muscle tension was clearly increased. Palpation did not touch obvious hepatosplenomegaly, and no murmur was heard in precordial auscultation. No other obvious abnormality was observed.

At birth, the total bilirubin was 15.7 mg/dl, direct bilirubin was 2.4 mg/dl, and indirect bilirubin was 13.3 mg/dl. Urinary GAGs, routine blood examination, blood gas, electrolyte, blood glucose, and thyroid function were all normal or negative. The anteroposterior chest X-ray showed small chest shape and short floating ribs. Both shoulder blades were small. The bilateral proximal humeri were irregular in shape (Figure 2C). X-ray of the pelvis anteroposterior showed deep cusp of large sciatic notch on both sides, small and shallow shape of the acetabulum on both sides, and lateral position of the bilateral femoral heads (Figure 2D). Echocardiography indicated mild tricuspid regurgitation without thickened valves. Cerebral ultrasound showed bilateral ventricle widening. Cranial MRI showed less white matter, smaller bilateral frontotemporal lobe, thinner corpus callosum, and delayed myelination (Figures 2E,F). Ultrasound showed that the liver and spleen were normal, without swelling. Superficial ultrasound suggested bilateral testicular hydrocele.

## Genetic Analysis

Case 1 was initially investigated as a genetic metabolic disease. Karyotyping of the patient was normal. No variant was found by mitochondrial DNA detection. Whole-exome sequencing (WES) found two heterozygous variants (c.1284+1G>T in intron 10 and c.1364C>T in exon 11 of the *GNPTAB* gene). The c.1284+1G>T, inherited from her mother, is a classical shear variant and may cause the splicing abnormality of mRNA that eventually affects protein function. The c.1364C>T, inherited from her father, is a rare missense variant (p.Aln155Val) and is located in a hot spot pathogenic area (Figures 3A,B). These two pathogenic variants have been reported previously, and bioinformatics analysis (e.g., PolyPhen2 and MutationTaster) suggested that they are harmful (6, 7). Variants c.1284+1G>T and c.1364C>T were, respectively, pathogenic and likely pathogenic according to the American College of Medical Genetics and Genomics (ACMG) criterion. The patient was finally diagnosed with ML III alpha/beta.

Case 2 was initially clinically suspected with a genetic metabolic disease. Subsequently, WES was undertaken, and two heterozygous variants (c.1284+1G>T in intron 10 and c.483delT in exon 5) of the *GNPTAB* gene were detected. The c.1284+1G>T was inherited from his mother. The c.483delT is a *de novo* pathogenic variant and causes frameshift, which leads to an obvious change of downstream amino acid sequence and premature termination (p.His162Ilefs^*^51). This variant was not found either in ExAC, GnomAD, or the 1000 Genomes database or in previous reports (Figures 4A–C). It was pathogenic according to the ACMG criterion. The patient was finally diagnosed with ML II.

## Discussion

ML II and III alpha/beta are two LSDs caused by variants in the *GNPTAB* gene. Nonsense or frameshift variants often cause complete loss of enzyme activity, leading to the more severe clinical phenotype, ML II alpha/beta. Missense and shear variants often retain partial enzyme activity, leading to the lighter clinical phenotype, ML III alpha/beta (8). The clinical manifestations of these two diseases are similar to those observed in some LSDs, such as MPS. However, it can be identified by urinary GAG screening, enzyme activity, and genetic analysis.

ML II, also known as I-cell disease, was first described in 1967 by Leroy et al. Its clinical symptoms are similar to those of Hurler syndrome. Analytically, it is characterized by a large number of inclusion bodies in a patient's fibroblasts and by negative results of urinary GAGs (9). The onset of ML II is relatively early, usually occurring during the perinatal period, and its symptoms are more severe. The growth of patients with ML II usually stops before 2 years of age, and their final height is usually <80 cm. Patients with ML II often present with progressive facial features, such as swollen eyelids, epicanthus, low-set ears, a protruding mouth, collapsed and wider nasal bridge, a short neck, rough and thickened skin, microcephaly, premature closure of cranial suture, and marked gingival hyperplasia. Patients with ML II frequently have dysostosis multiplex, limited joint range of motion, and motor retardation, often resulting in the inability to walk alone. The symptoms of most patients are accompanied by secondary hyperparathyroidism, which may be caused by abnormal placental calcium transport during the fetal period (10). Other clinical symptoms include language development retardation, mild to moderate intellectual disability, organomegaly, hypertrophic cardiomyopathy, recurrent respiratory tract infections, carpal tunnel syndrome, hernias, and obstructive sleep apnea (1, 11–13). Patients with ML II generally survive for <7 years. We compared the symptoms of case 2 with the typical symptoms of ML II. Case 2 presented with craniofacial dysmorphism, dysostosis multiplex, limited joint range of motion, motor retardation, language development retardation, hernia, mild to moderate intellectual disability, and recurrent respiratory tract infections. However, the patient did not show symptoms of organomegaly, hypertrophic cardiomyopathy, or obstructive sleep apnea. Hypertrophic cardiomyopathy and hepatosplenomegaly manifest after long-term survival. The absence of some of the clinical manifestations may be attributed to the short survival time of case 2. Unfortunately, we did not perform the nerve conduction study on case 2, so we could not confirm whether the patient had carpal tunnel syndrome.

The onset of ML III alpha/beta is relatively late, usually occurring in the early childhood, and the disease progresses slowly. Most patients show growth retardation after 3 years of age, with the final height often exceeding 115 cm, and relatively mild symptoms. The special facial features of ML III alpha/beta are relatively insignificant, with gradually appearing gingival hyperplasia and rough and thickened facial skin. Skeletal malformations in patients are milder than those of ML II. Patients are able to walk independently at an early stage, but they gradually lose the ability to walk or to move the joints with the onset of more severe symptoms. The language development process and intellectual development are mild or basically normal. In particular, patients with ML III alpha/beta have been reported to have tarsal tunnel syndrome (14). Other symptoms are similar to those of ML II, but milder. Patients often can survive to adulthood. Cardiopulmonary problems are the final cause of death in ML II and ML III alpha/beta (11). Case 1 presented with mild craniofacial dysmorphism, dysostosis multiplex, motor retardation, and recurrent respiratory tract infections. However, the patient did not develop the symptoms described above, such as gingival hyperplasia, rough and thickened facial skin, hepatosplenomegaly, and myocardial hypertrophy. This may be related to the younger age of case 1 and the slow progress of ML III alpha/beta. Moreover, case 1 had central hypothyroidism, which is not a common clinical manifestation of ML III alpha/beta. We speculated that this was due to the co-occurrence of these two disorders.

Imaging examination plays an adjunctive role in the diagnosis of ML. On X-ray, ML II often presents with dysostosis multiplex. The most common abnormalities in infancy and childhood are craniosynostosis, short anterior and posterior diameters of the thoracolumbar vertebrae, protuberance of the vertebrae, beak-like vertebrae, broad oar ribs with periosteum reaction, pelvic dysplasia, wrist dysplasia, severe delay of epiphyseal ossification, epiphyseal wear, and bone spot calcification, among others (5, 15, 16). Owing to a high-riding greater trochanter, the result of ultrasonography is often misunderstood as hip dislocation, which often leads to unnecessary correction (16). The X-ray imaging findings of ML III alpha/beta are similar when compared to those of ML II alpha/beta, but they are often lighter and develop more slowly. Ultrasonography revealed multifocal enlargement of the peripheral nerve in some patients with ML III alpha/beta (17). There are few reports on cranial computed tomography (CT) and MRI. In most cases, cranial CT shows brain atrophy, enlargement of the ventricles and subarachnoid space, and a decrease of white matter density. Brain MRI shows ventricular enlargement, frontal lobe atrophy and bifrontal leukomalacia, and delayed myelination or hypomyelination (18–23). We reported on two cases, both with dysostosis multiplex by X-ray and brain atrophy by cranial CT or MRI. Hence, ML should be considered in patients with dysostosis multiplex shown by X-ray and brain atrophy shown by cranial CT or MRI.

For patients with the above-mentioned clinical manifestations and imaging examination suspected with MPS, urinary GAG screening is often performed first. The DMB assay is the most commonly used detection method in hospitals, which often indicates negative results. Liquid chromatography–tandem mass spectrometry (LC-MS/MS) is an advanced method that suggests a slight increase in urinary GAG screening, which may provide support for better diagnosis in the future (24). The general increase of plasma lysosomal enzyme activity is a strong evidence of ML, but it is rarely measured in China. The discovery of pathogenic variants of the *GNPTAB* gene in genetic analysis is an important and a popular means for diagnosis because of the deficiency of enzyme activity detection in China. In the present manuscript, we reported on two patients with ML II and III alpha/beta. Case 1 had compound heterozygous pathogenic variants of the *GNPTAB* gene. The c.1364C>T variant was inherited from the father and the c.1284+1G>T variant inherited from the mother. The final diagnosis of this patient was ML III alpha/beta. Case 2 also had compound heterozygous pathogenic variants of the *GNPTAB* gene. The patient's variant loci were c.1284+1G>T, inherited from the mother, and c.483delT, a *de novo* variant. The patient was diagnosed with ML II alpha/beta. Among the three loci, c.1284+1G>T is a classic splicing variant, c.1364C>T is a rare missense variant, and c.483delT is a new frameshift variant reported for the first time in this paper. The final diagnosis of these two patients was consistent with the genotype. Including the two cases we reported, five patients with ML II and III alpha/beta were found to have the variant site (c.1284+1G>T), and all of them were Chinese.

Symptomatic treatment is applicable to almost all LSDs. For ML II and III alpha/beta, symptomatic treatment is the main treatment method and includes surgical treatment for hernia, orthopedic surgery for bone deformity, and heart valve replacement for severe heart valve disease, among others. Other special treatments, such as hematopoietic stem cell transplantation (HSCT), enzyme replacement therapy, and gene therapy, have also been studied. According to literature reports, although HSCT can correct some biochemical indexes, there is no evidence that it can significantly improve the clinical symptoms, neuromotor-related results, quality of life, or even the survival rate. In short, HSCT is not an effective treatment for ML (25–27). Although enzyme replacement therapy is now being used clinically in partial types of MPS, how to supplement various enzymes is still being studied owing to the lack of various lysosomal enzymes in the cells of ML patients (3). Gene therapy is a potential treatment, but no clinical research has been carried out in ML so far, so it cannot be applied in the clinic (3, 28).

## Conclusion

Here, we reported two cases of ML II and III alpha/beta caused by variants in the *GNPTAB* gene. Definitive diagnosis was achieved only after genetic testing through different NGS technologies, which revealed three different pathogenic variants (one novel and two previously reported). In summary, when patients have similar clinical manifestations, ML II and III alpha/beta should be considered in the differential diagnosis. Relevant examinations, such as the negative results of urinary GAGs, the increased multiple lysosomal enzyme activity in serum, and pathogenic variants in the *GNPTAB* gene detected by genetic testing, help in the diagnosis. Unfortunately, the parents of the two patients all rejected enzyme activity detection. This led to a defect in the diagnosis of the two cases. Presently, there is a lack of effective targeted treatment for these diseases. Symptomatic treatment is still the main treatment strategy. For pediatricians, prevention and treatment should focus on genetic counseling and prenatal diagnosis because of the deficiency of effective treatments.

## Data Availability Statement

The original contributions presented in the study are included in the article/supplementary material, further inquiries can be directed to the corresponding author.

## Ethics Statement

Written informed consent was obtained from the minor(s)' legal guardian/next of kin for the publication of any potentially identifiable images or data included in this article.

## Author Contributions

S-JM, Y-MZ, and Y-LD collected data and drafted the manuscript. C-CZ developed the research plan and revised the manuscript. All authors have read and agreed to the published version of the manuscript.

## Funding

This study was supported by the Key R&D Projects of Zhejiang Provincial Department of Science and Technology (2021C03094) and the National Natural Science Foundation (81371215 and 81670786). The funders had no role in the design of the study, in the collection, analyses, or interpretation of the data, in the writing of the manuscript, and in the decision to publish the results.

## Conflict of Interest

The authors declare that the research was conducted in the absence of any commercial or financial relationships that could be construed as a potential conflict of interest.

## Publisher's Note

All claims expressed in this article are solely those of the authors and do not necessarily represent those of their affiliated organizations, or those of the publisher, the editors and the reviewers. Any product that may be evaluated in this article, or claim that may be made by its manufacturer, is not guaranteed or endorsed by the publisher.

## Abbreviations

LSDs: lysosomal storage diseases
GAGs: glycosaminoglycans
MRI: magnetic resonance imaging
CT: computed tomography
HSCT: hematopoietic stem cell transplantation
MPS: mucopolysaccharidosis
ML: mucolipidosis
DMB: dimethylmethylene blue dye binding
LC-MS/MS: liquid chromatography–tandem mass spectrometry
WES: whole-exome sequencing
GlcNAc-phosphotransferase: *N*-acetylglucosamine-1-phosphotransferase.
